# Uncommon metastases of invasive lobular breast cancer to the endometrium: a report of two cases and review of the literature

**DOI:** 10.11604/pamj.2018.30.268.16208

**Published:** 2018-08-09

**Authors:** Raja Briki, Ons Cherif, Badra Bannour, Samir Hidar, Sassi Boughizane, Hedi Khairi

**Affiliations:** 1 Department of Gynecology and Obstetrics, Farhat Hached’s University Hospital, Sousse, Tunisia

**Keywords:** Invasive lobular breast carcinoma, endometrium, metastases

## Abstract

Although it is known that breast cancer can metastasize to many organ sites, metastasis to the uterus is uncommon and usually occurs during widespread metastatic disease. Lobular carcinoma is not the most common histological subtypes of breast carcinoma, but it is the most frequent histologic type that causes gastrointestinal, gynecological and peritoneal metastases. The main symptoms of the uterine metastasis depend on the anatomic involvement site. Abnormal uterine bleeding is by far the most important symptom.We highlight the importance of the follow up of patient with breast cancer. A rapid endometrial sampling for confirmation of the diagnosis, should be performed when the routine gynecological follow-up revealed any abnormality. We report two original observations of endometrium metastases of invasive lobular carcinoma of the breast which we detected during follow-up.

## Introduction

Breast cancer is the most common cancer in women, with a high mortality rate. Genital metastases of breast carcinoma are rare. Although lobular carcinoma is not the most common histological subtypes of breast carcinoma, it is the most frequent histologic type that causes gastrointestinal, gynecological and peritoneal metastases. The identification of unusual metastases in breast cancer drew particular attention to the evolution of invasive lobular carcinoma. There are now several publications that confirmed the risk of peritoneal, gynecological and digestive metastases of invasive lobular breast carcinoma [1, 2]. Uterine metastases account for approximately 4% of genital tract metastases, with 47% of cases involving the breast as the primary site [3, 4]. While there have been several case reports of metastases from the breast to the uterus, involvement of the endometrium by such metastases is extremely rare. We report two original observations of endometrium metastases of invasive lobular carcinoma of the breast.

## Patient and observation

**Figure 1:**
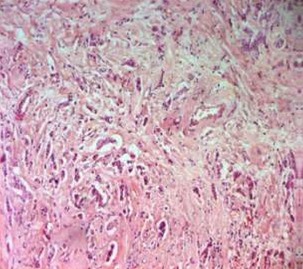
Invasive lobular carcinoma (HEx100)

**Figure 2:**
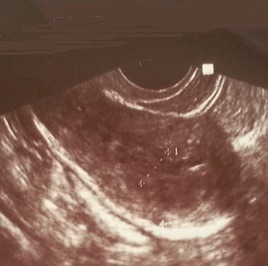
Transvaginal ultrasound revealed a thickened endometrium, similar to the increased endometrial proliferation associated with tamoxifen administration

**Figure 3:**
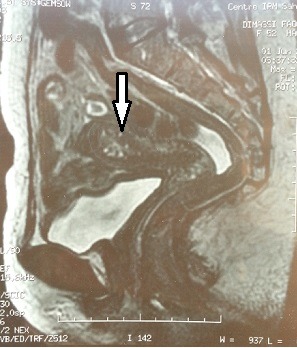
Pelvic magnetic resonance imaging revealed tumor infiltration of the endometrium less than 50% of the myometrium

**Case 1:** A 50-year-old woman was complaining of postmenopausal uterine bleeding. Her medical history revealed that she was diagnosed with breast carcinoma 2 years ago. At that time she enderwent a radical mastectomy associated with axillary lymph node dissection pathological examination of the tumor revealed invasive lobular breast carcinoma (Figure 1); stage IIb (T2 N1 M0). Immunohistochemical staining showed strongly positive for estrogen receptors (ER) and progesterone receptors (PR), negative for cerbB2. After surgery, she received adjuvant chemotherapy and left breast radiation. Since the tumor showed strongly positive estrogen receptors expression,we decided to put her under endocrine therapy treatment for 5 years. However, during the 23^th^ month of tamoxifen, she presented with postmenopausal uterine bleeding. Gynecologic examination was normal, pelvic ultrasound revealed that the endometrial thickeness was 13mm (Figure 2). Endometrial biopsy confirmed metastasis to the endometrium, from lobular breast carcinoma. Magnetic resonance imaging (MRI) revealed tumor infiltration of the myometrium less than 50% (stage IA as classified by FIGO 2009) (Figure 3). She underwent total abdominal hysterectomy and bilateral salpingo-oophorectomy. Breast carcinoma metastases in endometrium and myometrium were confirmed histopathologically and immunohistochemically.

**Case 2:** A 67-year-old woman was complaining of postmenopausal uterine bleeding. She has a medical history of hypertension and she is undergoing an hematologic exploration of bicytopenia. She was also diagnosed with bilateral breast carcinoma 3 years ago. She underwent a routine scintigraphy which revealed a single bony metastases on the spine treated with radiotherapy. At that time she underwent a left radical mastectomy and a right concervative breast cancer treatement (tumerectomy) associated with bilateral axillary lymph node dissection.Pathology was consistent with a multifocal infiltrating ductal carcinoma with 15 metastatic lymphnodes out of 16 at the left breast and an infiltrating ductal carcinoma measuring 1.5cm with no positif lymphnodes out of 15 at the left breast, ER/PR + and Her-2 Neu+. After surgery, she received adjuvant chemotherapy and bilateral breast radiation. Since the tumor showed strongly positive hormone receptors expression,we decided to put her under endocrine therapy treatment for 5 years. However, after 3 years, she presented with postmenopausal uterine bleeding. Gynecologic examination was normal, pelvic ultrasound revealed that the endometrial thickeness was 21mm. Endometrial biopsy confirmed metastasis to the endometrium, from lobular breast carcinoma. Magnetic resonance imaging (MRI) revealed stage Ib cancer as classified by FIGO 2009. She underwent total abdominal hysterectomy and bilateral salpingo-oophorectomy. Breast carcinoma metastases in endometrium and myometrium were confirmed histopathologically and immunohistochemically.

## Discussion

Metastases to the female genital tract from extragenital cancers are rare. Breast was the second most common primary site next to gastrointestinal [4]. Breast cancer includes a number of histological subtypes of which the two most common are invasive ductal carcinoma (IDC) and invasive lobular carcinoma (ILC). With respect to metastasis, ILC is the most frequent histologic type that metastasizes to the female genital tract in more than 80% of all cases [2, 5] . In patients with primary breast cancer maintained on Tamoxifen, there are few reported cases of uterine metastases [6-9]. Mazur et al reported that, among 149 metastatic tumors to the female genital tract from extragenital primaries, the ovary and vagina were the most frequent locations of metastases (75.8% and 13.4%, respectively) and only 8.1% were to the uterus [4]. Abnormal uterine bleeding is often the first symptom when the endometrium is involved. However, if the infiltration would affect myometrium only, patients may often be asymptomatic [7]. This case illustrates the importance of obtaining a rapid pathologic diagnosis, as treatment of Tamoxifen-induced endometrial primary disease and uterine metastases are very different [10]. There is a paucity of data regarding the best way to manage such cases. Given the limited number of reported cases, further studies are needed, along with a larger number of cases, to further our understanding of the prognosis of these uterine metastases and to determine the best course of treatment.

## Conclusion

We report two cases of metastatic breast lobular carcinoma involving tamoxifen to the endometrium. Uterine metastases of breast cancer are very rare; the presence of abnormal bleeding symptoms in a patient with a history of breast cancer should be suggestive of endometrium metastatic disease especially in case of ILC. We highlignt the fact that we should perform rapid endometrial sampling for confirmation of the diagnosis,when the routine gynecological follow-up revealed any abnormality.

## Competing interests

The authors declare no competing interest.
